# Semiconductor Nanocrystals as Light Harvesters in Solar Cells

**DOI:** 10.3390/ma6020445

**Published:** 2013-02-04

**Authors:** Lioz Etgar

**Affiliations:** Institute of Chemistry, Casali Institute of Applied Chemistry, The Hebrew University of Jerusalem, Jerusalem 91904, Israel; E-Mail: lioz.etgar@mail.huji.ac.il; Tel.: +972-2-658-5325

**Keywords:** quantum dots, heterojuction, Schottky, QD sensitized solar cell, organic-inorganic solar cell, photovoltaic

## Abstract

Photovoltaic cells use semiconductors to convert sunlight into electrical current and are regarded as a key technology for a sustainable energy supply. Quantum dot-based solar cells have shown great potential as next generation, high performance, low-cost photovoltaics due to the outstanding optoelectronic properties of quantum dots and their multiple exciton generation (MEG) capability. This review focuses on QDs as light harvesters in solar cells, including different structures of QD-based solar cells, such as QD heterojunction solar cells, QD-Schottky solar cells, QD-sensitized solar cells and the recent development in organic-inorganic perovskite heterojunction solar cells. Mechanisms, procedures, advantages, disadvantages and the latest results obtained in the field are described. To summarize, a future perspective is offered.

## 1. Introduction

To date, more energy from sunlight strikes the Earth in one *hour* (4.3 × 10^20^ J) than all the energy consumed on the planet in a *year* (4.1 × 10^20^ J). There is a huge gap between our present use of solar energy and its potential, which defines the grand challenge in energy research.

Currently, the photovoltaic field is divided into three generations. The first generation of solar cells refers to a single p-n junction of a crystalline Si (c-Si), exhibiting up to 25% conversion efficiency (lab), approaching the theoretical energy conversion efficiency (η) limit of 31% for single c-Si cell devices. This limit was predicted by a thermodynamic calculation of Shockley and Queisser (S-Q) for a photovoltaic conversion of solar irradiance in an ideal two level system [1]. The second generation of solar cells included the use of amorphous-silicon, poly-crystalline-silicon or micro-crystalline-silicon (a-Si, p-Si and mc-Si), cadmium telluride (CdTe) or copper (gallium) indium selenide/sulfide.

The third generation of solar cells, developed over the last decade, aims at conversion efficiencies beyond the S-Q limit of *η* = 31%. At the same time, their demands include the quality of the light absorbing materials, their arrangement and their $/KW-hour cost. The third generation solar cells are broadly defined as semiconductor devices; however, they differ from the previous generations in a few aspects: (a) First generation solar cells are configured as bulk materials that are subsequently cut into wafers and treated in a “top-down” method of synthesis (silicon being the most prevalent bulk material). Third generation solar cells are configured as thin-films (inorganic layers organic dyes and organic polymers), deposited on supporting substrates or as nanocrystal quantum dots (QDs) embedded in a supporting matrix in a “bottom-up” approach; (b) Third generation solar cells do not necessarily rely only on a traditional single p-n junction configuration for the separation of the photo-generated carriers. Instead, this generation includes the use of tandem cells, composed of a stack of p-n junctions of low-dimensional semiconductor structures. Within the limit of an infinite stack of a cascade with various *E*_g_, covering a wide range of the solar spectrum, the ultimate conversion efficiency at one sun intensity can increase to about 66%; (c) Third generation solar cells can be configured as donor-acceptor (D-A) hetero-junctions, with staggered electronic band alignment (named type-II configuration). These D-A devices include the photo-electrochemical cells, polymer solar cells and QD-solar cells.

Semiconductor quantum dots exhibit significant optical and electronic properties, which can be tuned according to their size. They are strongly luminescent [2], with various possibilities of preparation methods to control their size. It is clear that these semiconductor QDs are promising alternatives to molecular species for luminescence applications [2,3,4,5]. A wide variety of papers, reviews and books highlight the vast interest generated by the QDs [6,7,8,9,10,11,12,13].

Possible semiconductor QDs include CdS, CdSe, CdTe, CuInS_2_, Cu_2_S, PbS, PbSe, InP, InAs, Ag_2_S, Bi_2_S_3_,Sb_2_S_3_ and organo lead halide perovskite, which have been used as light harvesters in photovoltaic devices [14,15,16,17,18,19,20,21,22,23]. The short list of semiconductor QDs, which have been used as sensitizers in photovoltaic cells, presents the research areas that remain for researchers to explore for new semiconductors that can be used as light harvesters in QD-based solar cells.

This review focuses on QDs as light harvesters in solar cells, including different structures of QD-based solar cells—QD heterojunction solar cells, QD-Schottky solar cells, QD-sensitized solar cells and the recent development in organic-inorganic perovskite heterojunction solar cells. The mechanism, procedures, advantages, disadvantages and latest results obtained are described. In addition, a perspective on the future is offered.

## 2. Basic Terms for Photovoltaic Performance

**Figure 1:**
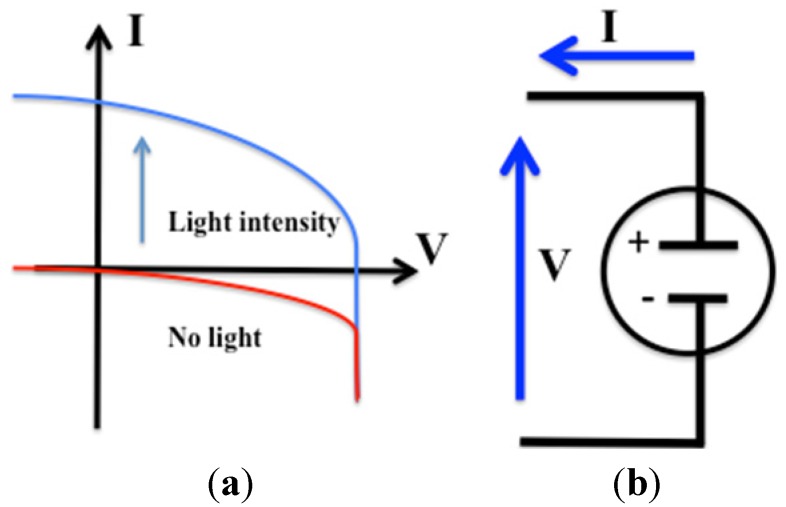
(**a**) I-V Curve of photovoltaic (PV) cell in darkness and under illumination; (**b**) Electrical diagram of a PV cell.

In general photovoltaic (PV) cells, can be modeled as a current source in parallel with a diode. As the intensity of light increases, current is generated by the PV cell. Where there is no light, the PV cell behaves like a diode (Figure 1).

The total current *I* in an ideal cell is equal to the current *I_l_* generated by the photoelectric effect minus the diode current *I_D_*, according to the equation:

(1) $$\text{I}={\text{I}}_{l}-I_{D}={\text{I}}_{l}-I_{0}\left(e^{\frac{qV}{kT}}-1\right)$$

where I_0_ is the saturation current of the diode, *q* is the elementary charge 1.6 × 10^−19^ Coulombs, *k* is a constant of value 1.38 × 10^−23^ J/K, *T* is the cell temperature in Kelvin and *V* is the measured cell voltage.

When taking into account the series and shunt resistances, equation 1 can be expanded to Equation (2), where *n* is the diode ideality factor (typically between 1 and 2) and *R_S_* and *R_SH_* represent the series and shunt resistances, respectively:

(2) $$\text{I}={\text{I}}_{l}-I_{0}\left(e^{\frac{q\left(V+IR_{S}\right)}{nkT}}-1\right)-\frac{V+IR_{S}}{R_{SH}}$$

When the voltage is equal to zero, the short circuit can be calculated (Jsc); The Jsc occurs at the beginning of the forward bias sweep. On the other hand, the open circuit voltage occurs when no current passes through the cell.

The solar cell is operated over a wide range of voltages (*V*) and currents (*I*). By continuously increasing the applied voltage on an irradiated cell, from *V =* 0 (with a short circuit current, *J_sc_*), through the point of *I =* 0 (with an open circuit voltage, *V_oc_*), to a very high value of *V*, it is possible to determine the maximum-power point at which the cell delivers maximum electrical power; thus, *V_m_ × I_m_ = P_m_*_ax_ in Watts. From that point on, the fill factor, defined as *FF = P_max_*/(*I_sc_V_oc_*)*,* is determined.

A larger fill factor is desirable and corresponds to an I-V sweep that is more square-like. Fill factor is also often represented as a percentage.

The power conversion efficiency (*η*), defined as the percentage of the solar power that is converted from absorbed light to electrical energy, is estimated [Equation (3)]:

(3) $$\eta =\frac{FF•V_{oc}•J_{sc}}{P_{in}}$$

where *P_in_* is the input light irradiance, which illuminates the cell. Additional details of the operation of solar cells and their principles can be found in review articles and textbooks [17,24,25,26].

## 3. QDs Solar Cell Structure

### 3.1. QD Heterojunction Solar Cells

In a heterojunction device, the top and bottom layers have different roles. The top layer, or *window layer*, is a material with a high band gap selected for its transparency to light. The window allows almost all incident light to reach the bottom layer, which is a material with low band gap that readily absorbs light. This light then generates electrons and holes very near the junction, which helps to effectively separate the electrons and holes before they can recombine.

**Figure 2:**
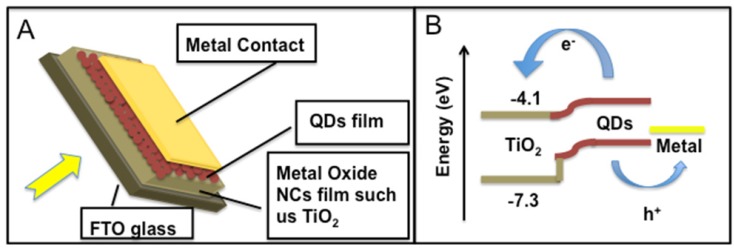
(**A**) Quantum dots (QDs) heterojunction solar cell; (**B**) Energy level diagram of QDs heterojunction solar cell.

In QD heterojunction solar cells, the bottom layer is composed of compact, mesocopic metal-oxide layers acting as electron collectors. Light is absorbed by the QDs with metal (usually gold or silver) as the top contact without additional electron blocking layers (Figure 2A). The conduction and valence bands of the QDs permit electron injection and hole transportation to the metal oxide and the metal, respectively (Figure 2B). The QDs in this cell structure are subsequently deposited layer by layer on the porous metal-oxide film by spin coating or dip coating of a concentrated QD solution. Each layer is cast at a high spinning rate or dipped in concentrated QD solution and then treated briefly with a solution of linker molecules intended to achieve dense and conductive QDs film. This treatment displaces the original ligand and renders the QD insoluble, allowing thin films of several hundred nanometer thicknesses to be created.

The number of QD layers deposited on the metal oxide plays an important role for the photovoltaic performance. If the QD layer is too thick, the collection of photogenerated charge carriers is incomplete, while too-thin QD layers show poor light harvesting.

There are several parameters that affect the photovoltaic performance in such a device architecture. The open circuit voltage, fill factor and photocurrent decrease with increasing the QD size; however, inter-particle electron transfer is facilitated in films made of the larger QDs, because for a given film thickness, there is a smaller number of particle boundaries to cross until the electrons arrive at the metal oxide. According to Matt Law and co-authors [18], the mobility of electrons and holes increases by one to two orders of magnitude with an increased QD diameter (e.g., a 2 nm increase in the QD diameter results in a one order of magnitude increase in the electron mobility). The size-mobility trends seem to be driven primarily by the smaller number of hops required for transport through arrays of larger QDs, but may also reflect a systematic decrease in the depth of trap states with decreasing QD band gap. These authors also observed that the carrier mobility is independent of the polydispersity of the QD samples. This fact is rationalized in terms of the smaller band gap, *i.e.*, larger diameter QDs carry most of the current in these QD solids if they can form a percolation network.

Recent investigations focus on depleted heterojunction devices, employing a mesoscopic wide band gap semiconductor oxide, such as TiO_2_ or ZnO, as a thin spacer layer between PbS QDs and the conducting transparent oxide current collector [19,27,28,29,30,31,32,33,34,35,36]. Efficiencies of 5%–6% were observed with these simple structures.

Sargent and co-authors [37] demonstrate the possibility of funneling energy toward an acceptor in QD heterojunction solar cells, involving a sequence of layers consisting of quantum dots selected as having different diameters and, therefore, different band gaps. The quantum funnel conveys photoelectrons from their point of generation toward an intended electron acceptor. This kind of solar cell benefitted from an enhanced fill factor. Another study by the same group discussed atomic ligands that make use of monovalent halide anions to enhance electronic transport and passivate surface defects in PbS QD films. Solar cells fabricated following this strategy show up to 6% solar AM1.5G power conversion efficiency [38]. Nozik *et al.* [39] introduce molybdenum oxide (MoO*_x_*) and vanadium oxide as a hole extraction layer in heterojunction ZnO/PbS quantum dot solar cells. They reported on power conversion efficiency of 4.4% certified by NREL. The hole extraction layer enhances the band bending to allow efficient hole extraction. The shallow traps in the MoO*_x_* layer enhance the carrier transport to the metal anode. The same researchers demonstrate, for the first time, superior stability of cells composed of ZnO NCs using air stable 1.3 eV PbS QDs. The stability was examined in a 1,000-hour test in air under constant illumination with no encapsulation applied to the device. The device demonstrates power conversion efficiency of 3% [40]. Etgar *et al.* presented for the first time the use of TiO_2_ nanosheets with 001 plane as the dominant exposed facet in PbS QD heterojunction solar cells, achieving a power conversion efficiency of 4.7% [33]. The better photovoltaic performance of the nanosheets compared to nanoparticles may be attributed to the higher ionic charge of the exposed (001) compared to the (101) facets, strengthening the attachment of the QDs to the TiO_2_ surface. Moreover, a detailed study on the electronic properties of heterojunction solar cells was made using electrochemical impedance spectroscopy [34].

A tandem heterojunction QDs solar cell has been demonstrated recently [31,41]. The tandem solar cell was made from different sizes of PbS QDs to increase the energy harvested from the sun. In order to allow the hole and electron to recombine, a graded recombination layer was used. The open circuit voltage of the tandem solar was about 1 V, which is the sum of the two constituent single-junction devices.

Finally, multiple exciton generation (MEG) was also witnessed in a similar QD-based solar cell structure [42,43]. The MEG effect requires a photon with an energy at least twice the band gap of the QDs; This produces two or more electron-hole pairs. Therefore, it is obvious that the MEG process can enhance the photocurrent of the solar cell. The authors of these reports observed external quantum efficiency exceeding 100%. This finding opens the way for enhancing the power conversion efficiency in QD-based solar cells beyond the S-Q limit of η = 31%.

### 3.2. QD-Schottky Solar Cells

Schottky-based solar cells are created from the Schottky junction between a semiconductor and a metal. Solar cells of this type have a long history, dating back to 1883, when Charles Fritts coated selenium with a thin layer of gold to make one of the world’s first solar cells.

**Figure 3:**
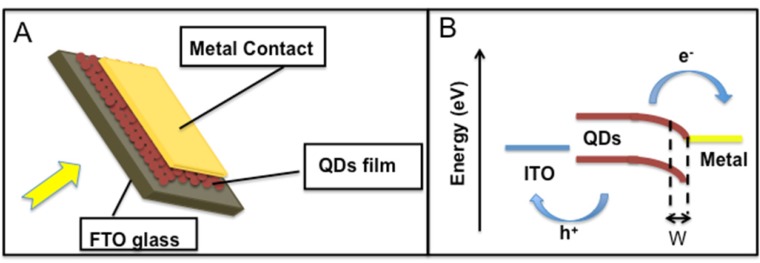
(**A**) QDs barrier Schottky solar cell; (**B**) Energy level diagram of QD barrier Schottky solar cell; W–width of the depletion layer.

How is a Schottky barrier created? When there is an interface between a metal and semiconductor, a depletion or inversion layer in the semiconductor is induced. A built-in potential, called the Schottky barrier, appears between the bulk of the semiconductor and the surface. The device architecture of a QD-Schottky barrier solar cell is shown in Figure 3A. The QDs are spin cast from solution, leading to smooth, densely packed arrays. The deposition techniques are similar to the one described for heterojunction solar cells. As shown in Figure 3B, a Schottky barrier is formed between the metal contact and the QDs film. Photogenerated holes are extracted through the transparent conducting ITO contact and a depletion region of width, W, forms near the Schottky contact.

An important factor in the QD-Schottky solar cell is the open circuit voltage (Voc), which increases proportionally to the band gap, described by: *V*_OC_ ≈ 0.49(*E*g/*q*) − 0.253 V, where *q* is the charge of an electron [44].

In a metal junction with semiconductor (p-type), the Voc of the cell decreases with the increased work function of the metal. However, Luther *et al.* [45] have found that the surface Fermi level can be pinned, so the barrier height is relatively independent of the metal.

Recent reports of QD Schottky solar cells using PbSe and PbS QDs show power conversion efficiencies (PCEs) of 1.8%–2.1% under AM1.5G illumination [46,47,48]. These results suggest that PbS and PbSe QDs films exhibit p-type semiconductor behavior after thiol treatment and form Schottky junctions on contact with metals.

QDs Schottky solar cells reach high short circuit current densities (Jsc), although in some cases, their open circuit voltage (Voc) remains low. For example, a Voc of ~0.05 V was obtained in a PbSe QD Schottky solar cell with an Au contact, due to the high work function of the Au [44]. As a result, air sensitive contacts of Ca or Mg metal coated with Al were required to increase the Voc of the Schottky junction (0.2–0.3 V of Voc) [46]. A further increase in QDs Schottky solar cell efficiency was reported recently, reaching a Voc of 0.51 V, introducing Al/LiF contact [49]. The increase in the Voc for QD Schottky solar cells, in addition to the high Jsc, puts them in a position to achieve higher efficiencies.

### 3.3. QD-Sensitized Solar Cells

**Figure 4:**
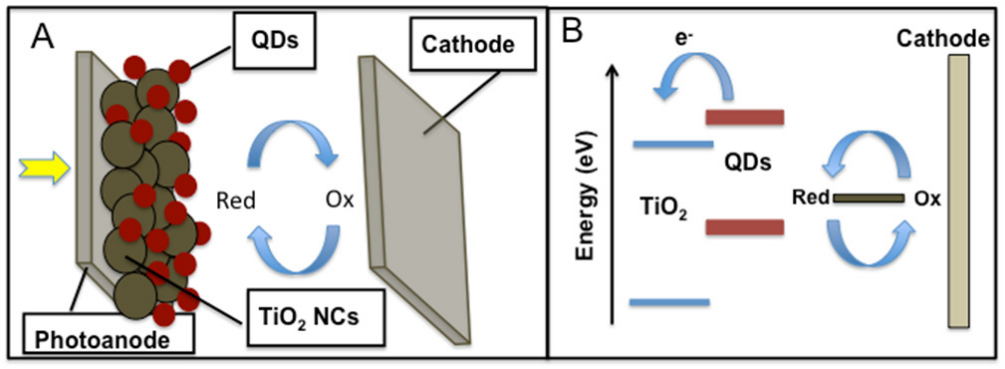
(**A**) The structure of a QD-sensitized solar cell; (**B**) Energy level diagram of a QD-sensitized solar cell.

Quantum-dot sensitized solar cells (QDSSC) are based on ensembles of nanometer size heterointerfaces between two semiconducting nanostructured materials. In this structure, QDs are attached to a wide band gap material (such as commonly used TiO_2_ or ZnO) through a linker with bifunctional molecules of the form X-R-Y for linking (where X and Y are functional groups, such as carboxylic, thiol *etc.*, and R is an alkyl group) or without a linker molecule, directly attached to the wide band gap material. Finally, a thin layer of liquid electrolyte containing a redox couple or a hole conductor (such as a hole conducting polymer) is sandwiched between this photoelectrode and a counter electrode to form the QDSSC. The device configuration depicted in Figure 4A separates the positive and negative photogenerated carriers into different regions of the solar cell using the following mechanism: after incident photons are absorbed by the QDs, photoexcited electron-hole pairs are confined within the nanocrystal. If they are not separated quickly, they will simply recombine.

After the electron is injected into the metal oxide, the positively charged QD can be neutralized either by hole injection into a hole conductor or through an electrochemical reaction with a redox couple in an electrolyte. The most common deposition techniques in QDSSC are the chemical bath deposition (CBD) and successive ionic adsorption and reaction (SILAR) process where the QDs attach directly to the wide band gap material. The CBD method is one of the cheapest methods to deposit thin films and nanomaterials. The CBD technique requires solution containers and substrate mounting devices. The chemical bath deposition yields stable, adherent, uniform, robust films with good reproducibility by a relatively simple process. The growth of the thin films strongly depends on growth conditions, such as duration of deposition, composition and temperature of the solution and the topographical and chemical nature of the substrate.

The SILAR process is based on sequential reactions at the substrate surface. Each reaction is followed by rinsing, which enables a heterogeneous reaction between the solid phase and the solvated ions in the solution. Accordingly, a thin film can be grown layer-by-layer, and the thickness of the film is determined by counting the deposition reactions. Examples of materials to be used in QDSSCs are CdS and CdSe nanocrystallites. These materials have shown the possibility to inject electrons to a wider band gap material, such as TiO_2_ [20,21,22,50,51], SnO_2_ [52,53] and ZnO [54,55].

Various semiconductor structures were tried in QDSSCs, such as alloys of CdSeS and core shells. Tunable energy band CdSe*_x_*S_(1−*x*)_ QDs were developed for QDSSCs by the SILAR technique. The results indicated that the energy band and the light absorption of CdSe*_x_*S_(1−*x*)_ QDs could be controlled by the ratio of the sulfur (S) and the selenium (Se), compared with the conventional CdS/CdSe system. The alloys system shows higher light harvest ability and a broader response wavelength region expressed by its absorption spectrum and IPCE spectrum. As a result, a power conversion efficiency of 2.27% was obtained with the CdSe*_x_*S_(1−*x*)_ QDSSCs under AM 1.5 illumination of 100 mW cm^2^. After being further treated with CdSe QDs, the CdSe*_x_*S_(1−*x*)_/CdSe QDSSCs yielded an energy conversion efficiency of 3.17% due to the enhanced absorption and the reduced recombination [56].

Recently, Zaban and co-authors [57] published a multilayer approach, consisting of multilayer CdSe QDs, which were assembled on a compact TiO_2_ layer. They showed that the sensitization of low-surface-area TiO_2_ electrodes with QD layers increases the performance of the solar cell, resulting in a 3.86% efficiency. The results showed the difference between dye-sensitized solar cells compared to QD-sensitized solar cells; when using a multilayer of dye molecules, the cell performance decreases, which is the opposite result of QD-sensitized solar cells. Further progress was achieved by Kamat *et al.* [58]. They demonstrate a 5.4% of power conversion efficiency by employing Mn^2+^ doping of CdS in QDSSCs. QDSSCs constructed with Mn doped CdS/CdSe were deposited on mesoscopic TiO_2_ film. The counter electrode in this study was Cu_2_S/graphene oxide, while the redox couple was sulfide/polysulfide. This cell showed good photostability for two hours under continuous illumination, achieving a steady photocurrent.

### 3.4. Organic-Inorganic Perovskite Heterojunction Solar Cells

**Figure 5:**
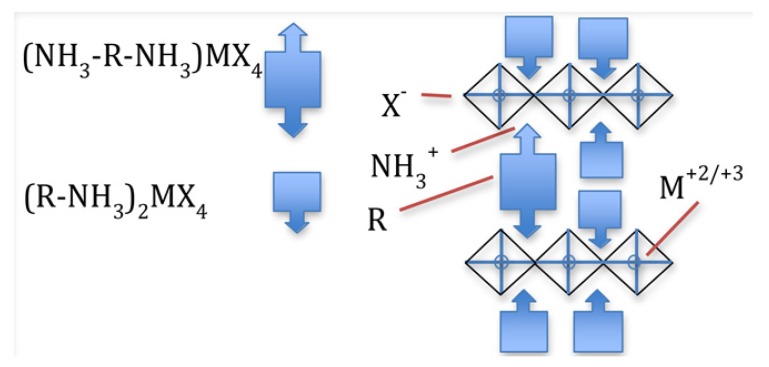
Single-layer oriented perovskites with monoammonium (R-NH^+3^) or diammonium (NH_3_^+^-R-NH^+3^) organic cations. Note that divalent (M^2+^) metals generally occupy the metal site.

The basic layered perovskite structures [59] are (R-NH_3_)_2_MX_4_ and (NH-R-NH )MX;(X = Cl^−1^, Br^−1^ or I^−1^), and they are schematically depicted in Figure 5. The inorganic layers consist of sheets of corner-sharing metal halide octahedra. The M cation is generally a divalent metal that satisfies charge balancing and adopts an octahedral anion coordination. Examples include Cu_2_^+2^, Ni_2_^+2^, Co_2_^+2^, Fe_2_^+2^, Mn_2_^+2^, Cr_2_^+2^, Pd_2_^+2^, Cd_2_^+2^, Ge_2_^+2^, Sn_2_^+2^, Pb_2_^+2^, Eu_2_^+2^ or Yb_2_^+2^. Recently, this family has been extended to include the trivalent metals, Bi_3_^+3^ and Sb_3_^+3^ [60].

The inorganic layers are usually called perovskite sheets, because they are derived from the three dimensional AMX_3_ perovskite structure, by making a one-layer-thick cut along the <100> direction of the three-dimensional crystal lattice. The structural modifications can be achieved by changing the compositions of the organic and inorganic salts in the starting solution to enable tailoring of the electronic, optical and magnetic properties.

The organic component consists of a bilayer or a monolayer of organic cations. In the case of the monolayer (monoammonium, as an example), the ammonium head of the cation bonds to the halogens in one inorganic layer, and the organic group extends into the space between the inorganic layers. For the bilayer (diammoniumcations, as an example), the molecules extend into the distance between the organic layers, which means that no van der Waals forces exist between the layers. The organic R-group most commonly consists of an alkyl chain or a single-ring aromatic group. These simple organic layers help define the degree of interaction between the inorganic layers and the properties developing in the inorganic layers. These important modifications are the result of changing the stoichiometry or composition of the organic and inorganic salts in the precursors solution used to grow the films or crystals. The layered perovskite described demonstrates that the inorganic sheets can determine the formation of single crystalline layers, which would achieve higher mobilities.

The direct band gap, large absorption coefficients [61] and high carrier mobility [62,63] of organo-lead halide perovskites present good potential for their use as light harvesters in mesoscopic heterojunction solar cells. Their electronic properties can be tailored, allowing for the formation of layered materials to control the distance and the electronic coupling between the inorganic sheets, according to the structure of the organic component employed. The layered perovskites have high stability in dry air. A few reports used CH_3_NH_3_PbI_3_ perovskite nanocrystals as sensitizers in photoelectrochemical cells with liquid electrolyte [23,64,65,66]. However, the performance of these systems rapidly declined due to dissolution of the perovskite. This problem was alleviated by replacing the electrolyte with a solid state hole conductor [66]. Very recently, the tin iodide-based perovskite CsSnI_3_ has been employed as a hole conductor, together with N719 as a sensitizer in solid state dye-sensitized solar cells, yielding a PCE of 8.5% [67]. Very recently, Snaith *et al.* [68] reported on efficient hybrid organic-inorganic solar cells, based on meso-superstructured organo halide perovskite, yielding a power conversion efficiency of 10.9%. This cell structure has few fundamental energy losses, so it can generate an open circuit voltage of more than 1V, despite the narrow energy gap (around 1.5 eV). The use of inert alumina oxide prevents the injection of electrons. As a result, the electrons are forced to reside in the perovskite and to be transported through it. In addition to this breakthrough, Etgar *et al.* [69] reported on the use of hole conductor free perovskite heterojunction solar cells. The authors found that the lead halide perovskite can transport holes, in addition to its functionality as an absorber, and achieved efficiency as high as 7% under low light intensity.

Table 1 lists various structures of QD-based solar cells, presenting the type of QDs used and their photovoltaic parameters. Due to the many publications available in this field, the table only includes a fraction of the results to highlight the cutting edge performance of each QD-solar cell structure.

**Table 1:** Summary of photovoltaic performance for various QD-based solar cell structures.

QDs Solar Cell Structure	QD Type	Jsc (mA/cm^2^)	Voc (V)	Efficiency (%)	Reference
Heterojunction	TiO_2_ NPs/PbS atomic ligands	20.2	0.48	6	[38]
Heterojunction	ZnO/PbS	18.1	0.524	4.4	[39]
Heterojunction	TiO_2_ nanosheets/PbS	20.5	0.545	4.7	[33]
Schottky	PbS	14	0.51	3.6	[45]
Schottky	PbS	24.5	0.239	2.1	[46]
Schottky	PbS_x_Se_1-x_	14.8	0.45	3.3	[49]
QDSSC (multilayer)	CdSe	12	0.556	3.86	[57]
QDSSC	Mn^+2^ doped CdS/CdSe	20.7	0.558	5.4	[58]
QDSSC	CdSe_x_S_(1− *x*)_/CdSe	12.27	0.44	3.14	[56]
Perovskite as hole conductor	N719	15.9	0.72	8.5	[67]
Perovskite sensitized solar cell	(CH_3_NH_3_)PbI_3_	17.6	0.88	9.7	[66]
Perovskite solid state solar cell	(CH_3_NH_3_)PbI_2_Cl	17.8	0.98	10.9	[68]
Hole conductor free perovskite solid state solar cell	(CH_3_NH_3_)PbI_3_	16.1	0.63	5.5	[69]

## 4. Future Perspective

Semiconductor QDs are promising alternatives to be used as light harvesters in solar cells. The properties of semiconductor QDs can be changed by tailoring their size. In addition, their band gap is tunable to different wavelengths of light, allowing them to harness energy from the visible to the infrared regions. QDs are inexpensive and easy to manufacture, making it possible to fabricate QD solar cells at low cost. There is room for major improvements in finding new semiconductors, which can be synthesized as QDs and function as light harvesters. This review shows possible architectures for QD-based solar cells, the influence of the cell structure on the cell mechanism and, hence, on the photovoltaic performance. Novel device architectures have much to offer the field, yet there is plenty of opportunity for further improvements through systematically engineering high-electron-mobility electrodes, such as nanopillars, nanowires and nanopores. The electronic interaction between QDs and electron acceptors is essential, and modifications of the photoanode surface will be required. The QD solar cells field has much to offer—devices with high performance, low fabrication cost and long-term stability can be expected in the future.
